# Prediction models for functional status in community dwelling older adults: a systematic review

**DOI:** 10.1186/s12877-022-03156-7

**Published:** 2022-05-30

**Authors:** Bastiaan Van Grootven, Theo van Achterberg

**Affiliations:** Department of Public Health and Primary Care, KU Leuven, Leuven, Belgium

**Keywords:** Prediction, Disability, Functional status, Community, Aged, ADL

## Abstract

**Background:**

Disability poses a burden for older persons, and is associated with poor outcomes and high societal costs. Prediction models could potentially identify persons who are at risk for disability. An up to date review of such models is missing.

**Objective:**

To identify models developed for the prediction of functional status in community dwelling older persons.

**Methods:**

A systematic review was performed including studies of older persons that developed and/or validated prediction models for the outcome functional status. Medline and EMBASE were searched, and reference lists and prospective citations were screened for additional references. Risk of bias was assessed using the PROBAST-tool. The performance of models was described and summarized, and the use of predictors was collated using the bag-of-words text mining procedure.

**Results:**

Forty-three studies were included and reported 167 evaluations of prediction models. The median c-statistic values for the multivariable development models ranged between 0.65 and 0.76 (minimum = 0.58, maximum = 0.90), and were consistently higher than the values of the validation models for which median c-statistic values ranged between 0.6 and 0.68 (minimum = 0.50, maximum = 0.81). A total of 559 predictors were used in the models. The five predictors most frequently used were gait speed (n = 47), age (n = 38), cognition (n = 27), frailty (n = 24), and gender (n = 22).

**Conclusions:**

No model can be recommended for implementation in practice. However, frailty models appear to be the most promising, because frailty components (e.g. gait speed) and frailty indexes demonstrated good to excellent predictive performance. However, the risk of study bias was high. Substantial improvements can be made in the methodology.

**Supplementary Information:**

The online version contains supplementary material available at 10.1186/s12877-022-03156-7.

## Introduction

Disability is a key outcome for public health [1]. It is associated with a decrease in quality of life, increased use of healthcare resources, institutionalization and mortality in community dwelling older persons [2], and is therefore generally considered a primary outcome for intervention (e.g. strength and mobility programs). The high prevalence of multimorbidity and the onset of functional impairments in older persons, make this population particularly vulnerable for the development of disability in their instrumental and basic activities of daily living (ADL), e.g. shopping, walking, washing [3–5]. Numbers vary substantially, but the incidence of disability in instrumental and basic ADL in community dwelling older persons ranges between 5% and 59% [6, 7]. The incidence is typically higher for instrumental ADL than for basic ADL [8], and also increases with age [9].

Decades of research have focused on identifying risk factors for disability in ADL in community dwelling older persons. Key risk factors include, among others, multi-morbidity, frailty, cognition, depression, body mass index, physical activity, and sensory and physical impairments [10–14]. If modifiable, these factors can be the focus of interventions. Alternatively, they can be used to identify an individuals’ risk for disability or predict a score on a disability scale when incorporated in a prediction model. Prediction models can inform persons about their individual prognosis (risk), can support older persons and healthcare professionals in the decision-making process, and can inform research designed to explore subgroups that respond better (or worse) to interventions that aim to improve functional status or prevent disability.

One systematic review, published in 2015, has previously investigated the utility of clinical prediction models for the outcome functional decline [15]. This review included 16 models in the evaluation and observed areas under the curve ranging between 0.63 and 0.78, thus indicating poor to moderate predictive performance of the models. However, this review had some limitations, e.g. only including short case finding instruments, only including instruments with validation, only considering decline in ADL as outcome. Also, a large number of studies have since been published. A second review investigated the association between frailty indicators and disability, and observed an important association between gait speed and disability [9].

We therefore performed a systematic review to identify models developed for the prediction of functional status in community dwelling older persons. We investigated the types of predictors that were included in the models, and how well the models predicted functional status.

## Methods

A protocol for the systematic review was drafted before the start of the study (see appendix 1) and the PRISMA statement was used to structure the report of the study. [16]

### Eligibility criteria

Studies had to include older persons, indicated by a mean sample age of 65 years or older, with the majority of the sample living at home.

Prediction models described in the studies had to predict the outcome functional status, defined as the ability to perform instrumental or basic ADL, and could include ADL scales (e.g. Katz scale) or specific aspects of ADL (e.g. washing or mobility). Physical performance outcomes were considered relevant if the reported data related to daily activities, e.g. ability to mobilise. Physical performance related to strength or speed was not considered for inclusion.

Predictions could include the prediction of ADL (scale or item score), or decline, maintenance, recovery or improvement in ADL (scale or item). The prediction model had to measure a single characteristic (univariable model) or a set of characteristics (multivariable model) to estimate a person’s individual prognosis and could include patient, care outcome and care process factors. The models could be presented in any format, e.g. as a statistical model, regression formula with coefficients, web or electronic application, nomogram, or score chart.

Studies with binary or survival outcomes had to report the concordance (c) statistic (which is equal to the area under the curve). Studies with continuous outcomes had to report the R2 statistic.

We included nested case-control studies, prospective and retrospective cohort studies (including database and registry studies), and secondary analyses of trials.

### Information sources

We searched the Medline and EMBASE databases from inception up to March 2022 for eligible studies. After selecting the full text manuscripts, we screened references lists and prospective citations (using Google Scholar) for eligible studies. Lastly, the ICTPR portal was searched for protocols and Web of Science for conference proceedings in order to track full text manuscripts.

### Search

A search string was drafted using a combination of free text words and MeSH terms. The search terms were grouped according to outcome, prediction models, and setting. We used a validated search string for the terms related to the prediction models [17]. The terms related to the outcome and setting were derived from other systematic reviews, entry terms related to MeSH term, thesaurus searches for synonyms, and key words from published manuscripts. The final search string was adapted to the EMBASE database. No limits were used for the search. The final search string is available in appendix 2.

### Study selection

Identified records were collected in an Endnote database. One author screened all titles and abstracts for inclusion in two stages: screening titles and abstracts, and reading full text manuscripts. The second author verified the final inclusion using a standardised checklist. The final selection was based on a consensus decision. Authors were not blinded to the manuscripts’ citation information.

### Data collection process

Data were collected in an Excel database. The data collection was first piloted in order to standardise the collection process and define uniform terms. The data collection was performed by one author. Data were verified a second time by the same author.

### Data items

The following information was collected: study citation, country, design, setting, sample characteristics, sample size, outcome definitions and measurements, statistical analyses, definition of predictors, purpose and design of prediction models, and the evaluation and performance of prediction models.

### Risk of bias

The PROBAST tool (Prediction model Risk Of Bias ASsessment Tool) was used to assess the risk of bias for the participants, predictors, outcome and analysis for each model [18]. A standardised questionnaire was used to rate the risk of bias as ‘yes’, ‘probably yes’, ‘no’, ‘probably no’, or ‘no information’. An overall judgement was made as either low risk of bias, high risk of bias or unclear risk of bias. One author assessed the risk of bias.

### Summary measures

We investigated the discrimination and calibration for models with a binary and survival outcome, and the R2 measure and calibration for models with a continuous outcome [19]. The discrimination was expressed using the concordance (c) statistic. The c statistic is equivalent to the area under the curve (for binary outcomes), and the following values were used in the interpretation of the performance: < 0.7 is poor, 0.7 – 0.8 is moderate, 0.8 – 0.9 is good, > 0.9 is very good [20]. The performance of linear models was measured using R2. The calibration could be measured using different statistics: observed versus expected events, calibration slope, calibration in large, calibration plot, or the Hosmer-Lemeshow test.

### Synthesis of results

Summary tables were drafted to describe the study characteristics, risk of bias and findings of the studies. We originally planned to construct funnel plots to visualize the different performance measures (R2, discrimination, calibration). However, the majority of the studies did not report standard errors or sufficient information to construct confidence intervals. We changed our strategy and described the distribution using scatter plots to visualize the discrimination and R2 of the individual studies, and constructed box plots to describe the central tendency (median) and spread of the data (interquartile range). We further observed substantial differences between studies and models and therefore decided to describe the results within subgroups based on the 1) definition of the outcome (ADL, IADL, ADL or IADL, mobility or disability), 2) type of summary measure (c statistic or R2), 3) type of model (univariable or multivariable), 4) type of model evaluation (development or validation). We used the ‘bag of words’ text mining procedure to summarise the clinical predictors included in the models. This procedure was used to create a word cloud to describe the frequencies of the predictors. Analyses were performed using R studio (using the ‘dplyr’, ‘ggplot2’, ‘Hmisc’, ‘tm’ and ‘wordcloud’ packages).

In addition, we described individual models that demonstrated a good performance in a validation cohort (c statistic > 0.8, R2 > 0.5) separately using a narrative synthesis.

## Results

**Fig 1.**
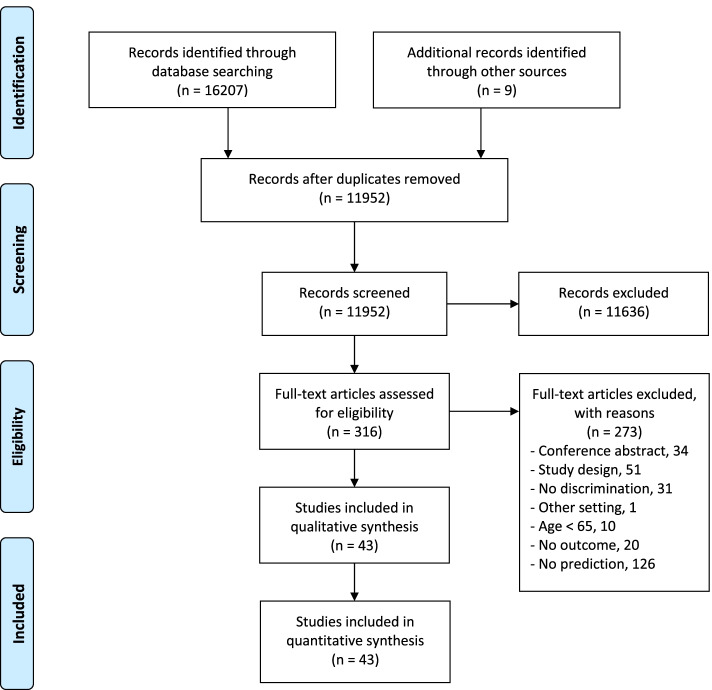
Flowchart

A total of 11952 titles and abstracts, and 316 full text manuscripts were screened for inclusion. A total of 34 studies were retained for inclusion. [6, 7, 21–52] An additional nine studies were identified through secondary sources [53–61], resulting in a final sample of 43 studies (see Figure 1).

### Study characteristics

The majority of studies originated from North America (*n* = 19) or Europe (*n* = 15), and to a lesser extent from Asia (*n* = 7), or South America (*n* = 1) or Africa (*n* = 1). All but one study used data from a prospective cohort study; the one study using data from a randomized controlled trial. The median age across the samples was 76 years, with a minimum of 66 and a maximum of 85. Eighteen (43%) of the studies recruited persons who were independent in ADL or mobility at baseline. Basic ADL was the most prevalent outcome (*n* = 15), followed by Instrumental ADL (IADL, *n* = 8), mobility (*n* = 5), disability (*n* = 4), or a composite outcome of ADL and IADL (*n* = 4). The remaining studies had multiple outcome measurements of ADL, IADL, mobility, or bathing and dressing. Almost all studies evaluated predictions of functional decline (*n* = 39), two studies predicted a change in outcome score, and two studies predicted the outcome score on a continuous scale. The median incidence of functional decline across the studies was 20%, with a minimum of 5% and a maximum of 59%. The median time to follow-up across the studies was two years, with a minimum of half a year and a maximum of nine years. The 43 studies included 167 evaluations of prediction models. The results will be reported at the level of the 167 evaluations of prediction models. The characteristics are described in table 1.

**Table 1.** Study characteristics

**Author**	**Year**	**Country**	**Design**	**Population**	**Age** ^a^	**Aim**	*n*	**Outcome, FU** ^b^
**Adachi** [17]	2018	Japan	Prospective cohort	Older people walking independently	79 [76 - 82]	Evaluate factor	518	Mobility limitation, 2
**Adachi** [18]	2018	Japan	Prospective cohort	Women 75 or older walking independently	79 [77 - 82]	Evaluate factor	330	Mobility limitation, 2
**Aliberti** [19]	2018	USA	Prospective cohort	65 or older without dementia or ADL dependence	74.4 (7.0)	Develop model Validate index	7388 7388	ADL dependence, 2,
**Arnau** [20]	2016	Spain	Prospective cohort	75 or older without severe dependence	81.7 (4.6)	Develop model	252	IADL or ADL decline, 1
**Ben-Shalom** [21]	2016	USA	Prospective cohort linked with administrative database	Medicare beneficiaries 65 or older		Develop model	10057	ADL dependence, 4
**Bongue** [48]	2017	Canada	Prospective cohort	65 or older	78.7 (7.9)	Validate index	1224	Disability, 2
**Brach** [22]	2012	USA	Prospective cohort	65 or older walking independently	79.4 (4.1)	Evaluate factor	339	Mobility limitation, 1
**Carrière** [49]	2005	France	Prospective cohort	75 or older independent in IADL	79 [76 - 81]	Develop model Validate model	545 807	IADL dependence, 7
**Clark** [50]	2012	USA	Prospective cohort	65 or older independent in ADL	74.4 (7.2)	Develop model Validate model	6233 3213	ADL dependence, 2
**Clark** [6]	2015	USA	Prospective cohort	65 or older independent in ADL	74.1 (6.7)	Develop model Validate model	5332 2763	ADL dependence, 2
**Classon** [23]	2016	Sweden	Prospective cohort	85 or older		Evaluate factor	83	IADL decline, 5
**Covinsky** [24]	2006	USA	Prospective cohort	70 or older independent in ADL	76.8 (5.3)	Develop model Validate model	3245 1994	ADL dependence, 2
**Deckx** [7]	2015	Netherlands	Prospective cohort	70 or older with and without cancer	78.1 (5.5)	Validate index	134 220	ADL decline, 1
**Dixon** [48]	2021	USA	Prospective cohort	65 or older	75.4 (6.1)	Develop model	93	IADL dependence, 1.5
**Donoghue** [25]	2014	Ireland	Prospective cohort	50 or older	72.8 (6.1)	Evaluate factor	1664	ADL dependence, 2 IADL dependence, 2
**Ensrud** [51]	2009	USA	Prospective cohort	67 or older men walking independently	76.4 (5.6)	Validate index	3132	IADL dependence, 3.2
**Faurot** [26]	2015	USA	Prospective cohort linked with administrative database	65 or older		Develop model	6391	ADL dependence, 4
**Gill** [27]	1997	USA	Prospective cohort	72 or older independent in ADL	78.5 (5.2)	Validate index	1813	ADL dependence, 1
**Gobbens** [52]	2012	Netherlands	Prospective cohort	75 or older	80.3 (3.8)	Update index	479	Disability, 2
**Guralnik** [53]	2000	USA	Prospective cohort	65 or older without disability		Evaluate factor	6534	Disability, 1-4
**Hegendorfer** [28]	2019	Belgium	Prospective cohort	80 or older	84.7 (3.7)	Validate index	560	ADL decline, 1.7
**Hong** [29]	2016	South Korea	Prospective cohort	60 or older	72.5 (55)	Evaluate factor	8000	IADL decline, 3
**Ishimoto** [30]	2010	Japan	Prospective cohort	65 or older	75.5 (5.9)	Validate index	518	ADL decline, 1
**Jonkman** [31]	2019	Germany, United Kingdom, Italy, Netherlands	4 Prospective cohorts	65 - 75y at baseline without limitations	69.7 (3.0)	Develop model Validate model	2560 2560	ADL dependence, 3
**Jonkman** [32]	2018	Italy, Netherlands	2 Prospective cohorts	60 - 70y at baseline	67.5 (2.1)	Develop model	312	IADL or ADL decline, 9
**Lam** [33]	2020	Hong Kong	Prospective cohort	65 or older walking independently	72.5 (5.3)	Validate index	1566	Physical limitations, 4
**Lin** [34]	2004	Taiwan	Prospective cohort	65 or older	73.4	Evaluate factor	1200	ADL decline, 1
**McClintock** [35]	2018	United States	Prospective cohort	Medicare beneficiaries 65 or older		Develop model Validate model	12758 8506	IADL decline, 2
**Nuesch** [36]	2015	UK	2 Prospective cohorts	Older adults without locomotor disability	61 - 68	Develop model Validate model	2377 3194	Locomotor disability, 7-8
**Onder** [54]	2005	USA	Prospective cohort	Older women with functional disability	78.7 (8.0)	Evaluate factor	484 684	ADL dependence, 3 Mobility limitation, 3
**Op het Veld** [55]	2019	Netherlands	Prospective cohort	65 or older who are (pre)frail	76.3 (6.6)	Validate index	2420	IADL or ADL decline, 2
**Papacristou** [37]	2017	UK	Prospective cohort	Men between 71 - 92	8.0 (4.4)	Evaluate factor	1198	Mobility limitation, 3
**Perera** [38]	2015	International	7 Prospective cohorts	Older persons	73.9 (5.3)	Evaluate factor	27220	Bathing/dressing dependency, 3 Mobility limitation, 3
**Saraiva** [39]	2020	Brazil	Prospective cohort	60 or older	80 (12)	Validate index	317	ADL decline, 1
**Sarkisian** [40]	2000	USA	Prospective cohort	Women 65 or older	73	Develop model Validate model	4421 2211	IADL decline, 4
**Spalter** [41]	2013	Israel	Prospective cohort	60 or older	70.9	Develop model	982	Change in mobility, 5
**Studenski** [56]	2003	USA	3 Prospective cohorts	65 or older	74.1 (5.7)	Evaluate factor Validate index Develop model	974	IADL or ADL decline, 1
**Suijker** [42]	2014	Netherlands	Prospective cohort	70 or older	76.1 [8.4]	Update index Validate index	644 2085	ADL decline, 1
**Tas** [43]	2011	Netherlands	Prospective cohort	55 or older	> 65	Develop model	5027	Mild disability, 6
**Teo** [44]	2017	Signapore	Prospective cohort	55 or older independent in ADL	66.1 (7.6)	Validate index	2406	IADL dependence, 2 ADL dependence, 2
**Terhorst** [45]	2017	USA	Prospective cohort	Women 70 or older	78.9 (5.1)	Develop model	256	Mobility limitation, 0.5 ADL dependence, 0.5 IADL dependence, 0.5
**Wennie Huang** [46]	2010	USA	Prospective cohort	65 or older independent in ADL	80.3 (7.0)	Evaluate factor	110	ADL dependence, 0.5 – 1.5
**Yam** [47]	2013	USA	Secondary analysis of RCT	65 or older	-	Develop model	582	IADL function, 5

*ADL* Activities of Daily Living, *IADL* Instrumental Activities of Daily Living, ^a^ data is reported as mean (standard deviation) or median [interquartile range]; ^b^ FU = Follow-up, is reported as number of years.

### Risk of bias

**Fig 2.**
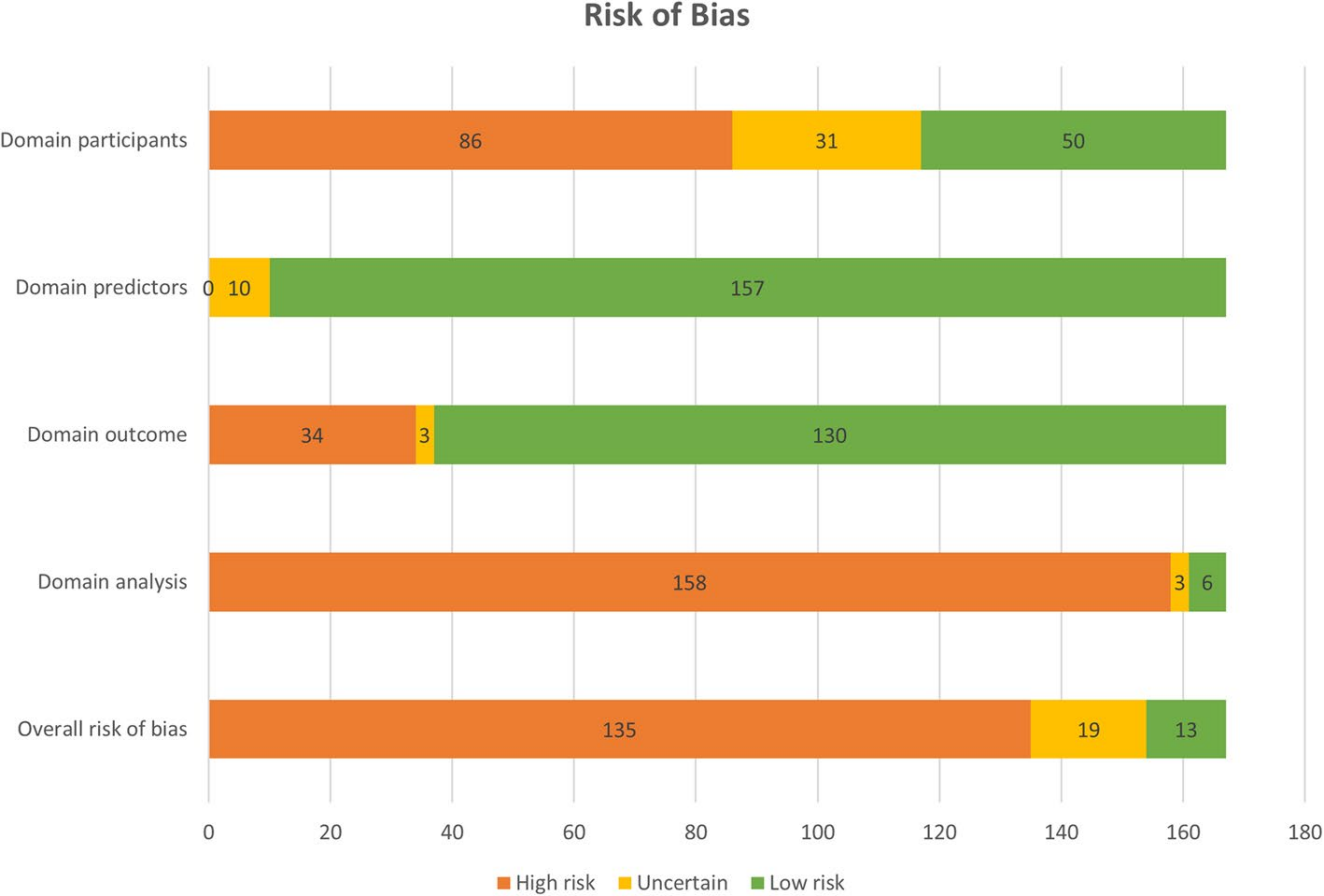
Risk of bias

There was a low risk of bias in 13 model evaluations, high risk of bias in 135 evaluations, and there was uncertainty in the remaining 19 evaluations (see Figure 2). The majority of the model evaluations had a low risk of bias on the domains related to measuring predictors (*n* = 157) and outcomes (*n* = 130). However, the majority of evaluations had a high risk of bias on the domains related to recruiting participants (*n* = 86) and analyses (*n* = 158). A clear description of the recruitment was often missing or only participants with complete data were selected from the cohort for analysis. Predictors were often selected based on p-values without accounting for overfitting or optimism in the performance, and the influence of right censoring (e.g. due to death) was not estimated.

### Prediction models

Of the 167 evaluations, 62 were univariable model evaluations, of which 58 estimated the risk for functional decline, and four validated previously determined cut-off criteria. Sixty-seven evaluations of multivariable models were performed, of which twelve were also validated by the authors who developed the model. Thirty-eight multivariable model evaluations were external validations by an independent research team. The median sample size across the models was 1198, with a minimum of 83 and a maximum of 27220.

### Performance of models

**Fig 3.**
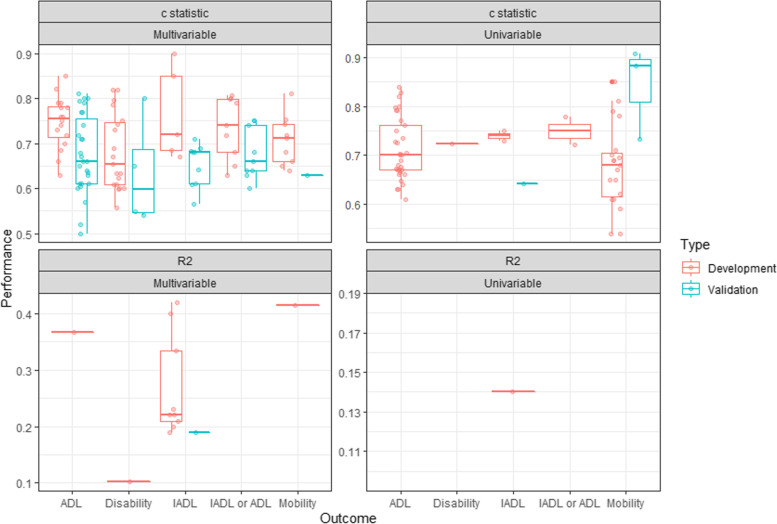
Performance of models

In the evaluations (see Figure 3), the median c statistic values for the multivariable development models ranged between 0.65 and 0.76 (minimum = 0.58, maximum = 0.90), and were consistently higher than the values of the validation models for which median c statistic values ranged between 0.6 and 0.68 (minimum = 0.50, maximum = 0.81). The values of the univariable models tended to be similar to those of the multivariable models (median values ranging between 0.68 and 0.75 (minimum = 0.54, maximum = 0.85) for development, and ranging between 0.64 and 0.89 (minimum = 0.64, maximum = 0.91) for validation).

The R2 values of the multivariable development models ranged between 0.10 and 0.42, but only the outcome IADL had sufficient observations to estimate the distribution parameters. The performance of the multivariable models appeared to be similar for the different outcomes. The calibration was measured in nine evaluations. Six evaluation reported the expected versus observed events, two used a calibration plot, and one evaluation estimated the calibration slope and intercept.

Four models demonstrated a good performance in a validation cohort. However, the risk of bias was high in the first three models, and uncertain in the last model. Clark et al. included nine baseline predictors in a model to predict ADL dependence and validated the model at two years follow-up (c statistic = 0.80) [6]. Gobbens et al. evaluated the Tilburg Frailty Index (15 predictors) for the prediction of disability measured using the Groningen Activity Restriction index at two years follow-up (c statistic = 0.8) [57]. Ishimoto et al. evaluated a 21-item fall risk index for the prediction of ADL decline at one year follow-up (c statistic = 0.8) [34]. Teo et al. validated a social and physical frailty index (18 predictors) for the prediction of severe ADL disability at half a year follow-up (c statistic 0.81) [48].

Two univariable models demonstrated a good to excellent performance in a validation cohort. However, the risk of bias was uncertain for both models. Both models were evaluated in the same cohort by Adachi et al., which observed that a gait speed of <0.8m/s had an excellent discrimination (c statistic 0.91), and a gait speed of <1.0m/s had a good discrimination (c statistic 0.89) for the outcome mobility limitations at two years follow-up [22].

### Predictors

The median number of predictors in the multivariable models that were evaluated was seven, with a minimum of two and a maximum of 26. Six model evaluations included longitudinal repeated measurements of predictors, with the remaining models only included baseline measurements.

**Fig 4.**
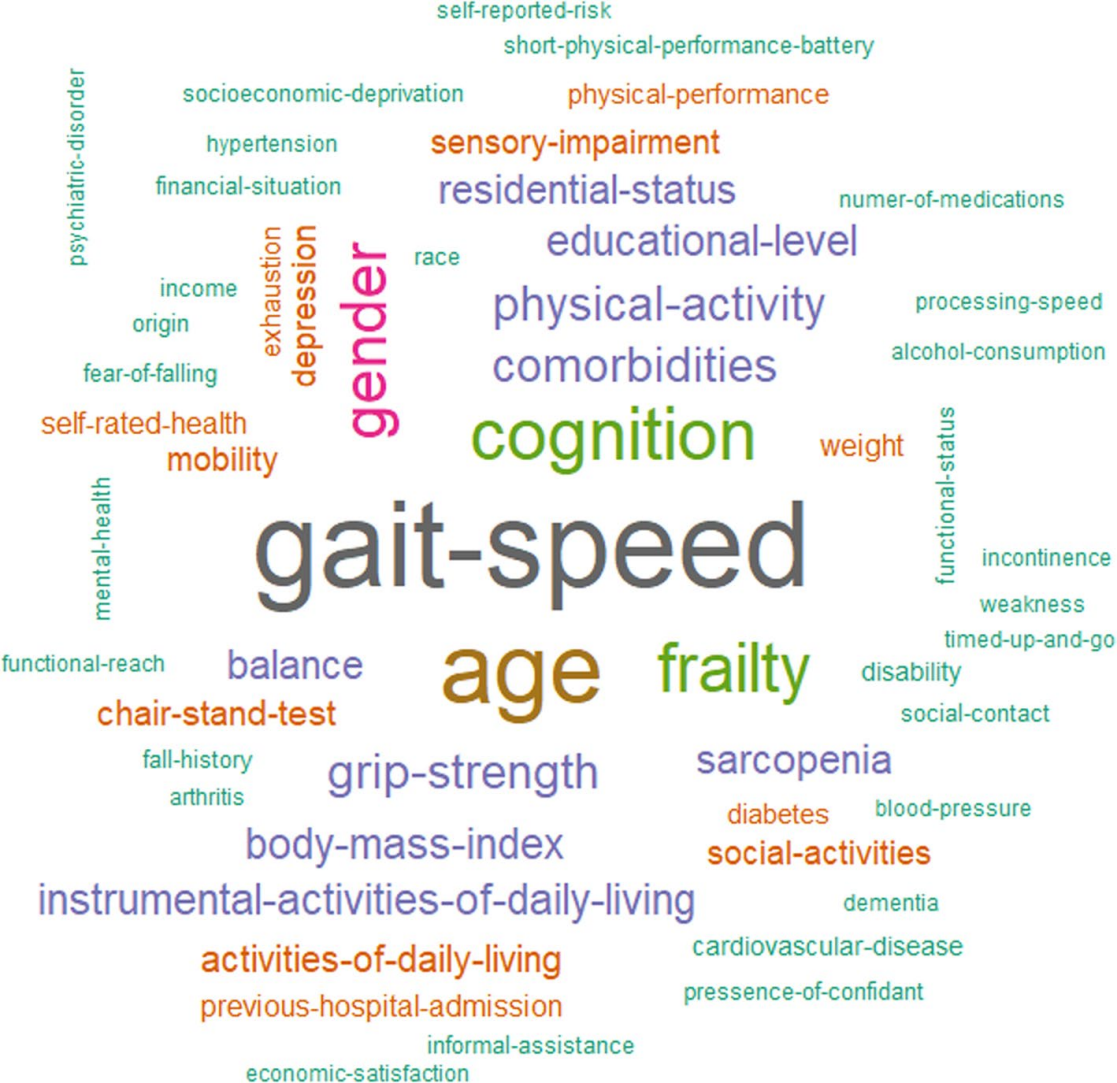
Predictors in prognostic models

A total of 559 predictors were recorded, with 55 predictors being used in at least three evaluations (see Figure 4; note that the larger words indicate a higher frequency of use of the predictor in the models). The fifteen predictors most frequently used were gait speed (*n *= 47), age (*n* = 38), cognition (*n* = 27), frailty (*n* = 24), gender (*n* = 22), comorbidity (*n* = 15), grip strength (*n* = 15), physical activity (*n* = 15), body mass index (*n* = 15), IADL (*n* = 13), balance (*n* = 12), educational level (*n* = 12), residential status (*n* = 12), sarcopenia (*n* = 12), and ADL (*n* = 10).

## Discussion

This review evaluated the state of the art for models that aim to predict future functional status in older persons. We identified the models, and summarised their performance, as well as the predictors that were used. Self-reported (in)dependence on activities of daily living was the most prevalent outcome for the prediction of functional status. The performance of the models varied substantially, ranging from poor to very good, but was moderate to low on average. The performance of the prediction models was generally lower in validation cohorts. 

Multivariable models appeared to be slightly better for the prediction of future functional status than univariable models, but this was difficult to assess because most univariable models lacked external validation. Gait speed was the most prevalent predictor, in particular for univariable predictions, and demonstrated a moderate predictive performance (median c statistic = 0.70, minimum = 0.64, maximum = 0.91, data not shown). The majority of studies (95%) did not evaluate the calibration of the model.

Our results are in line with the previous review on prediction models for community dwelling older adults [9, 13], that also observed a poor to moderate performance of prediction models for the outcome functional decline. Our review included more and different outcomes, but conclusions are the same across the different outcome measurements. We also included substantially more and more recent models, but the state of the art does not appeared to have improved. To date, no model appears to be ready for implementation in clinical practice. However, frailty models and gait speed measurements appear to be the most promising. Nonetheless, the high risk of bias for many studies, including those on frailty models, is a particular concern. Most studies ignored missing data and censoring of outcomes, which can bias the regression coefficients and ultimately lead to poorly calibrated models at the population level. Furthermore, calibration was only investigated in 5% of the models. Nonetheless, this is a key measure for prediction models as it assesses if the predicted outcome (probability or score) corresponds to the observed outcome. Poorly calibrated models over or under predict the probability and can potentially harm the subsequent decision-making process [62].

Specific suggestions can be made to improve the body of evidence on the prediction of functional status. The majority of studies relied on statistical significance to select predictors, which can result in overfitting and overly optimistic performance measures for the developed model [63]. This optimism is often not detected because most models are never validated. This could be mitigated by finding consensus on a core set of prognostic factors for functional status. For example, stacked regression could be used to derive predictors from a combination of different prediction models, i.e. the analysis would find the ideal combination of predictors across the models [64]. If new predictors are tested, and if testing relies on statistical selection, than penalised regression models, e.g. lasso-regression, is preferred; or a shrinkage factor should be applied to minimise the optimism as a result of predictor selection. These methods were not used in any of the included studies. An important discussion should also be the appropriate selection of statistical methods. The predominant method, logistic regression, does not account for censored observations (e.g. due to death) and disregards the missing data. Furthermore, the design of cohort studies have an inherent risk for attrition bias, i.e. that patients who experience disability or die are also more likely to have missing values because they drop-out of the study. The association between disability and death is worrisome, because it is conceivable that persons who have died would have experienced a different disability trajectory than persons who survived. A model that ignores this will have biased coefficients and therefore predictions [65]. These ‘informative right censoring’ assumptions should at least be tested in samples with loss to follow-up, e.g. using a joint model strategy. Lastly, clinical usefulness should also be part of the evaluation of a model that is well calibrated. Classification plots with the area under the curve and classification measures (e.g. sensitivity, specificity) for different potential cut-off values should be preferred over ROC curves [66].

The results of this review are somewhat discouraging. Nonetheless, the burden of disability remains high and will be an important driver for increasing long-term healthcare costs [67]. We believe that the identification of persons who could benefit from interventions designed to prevent disability therefore remains a worthwhile public health strategy.

Lastly, it is important that the impact of models is evaluated in practice. We have additionally searched for studies that implemented disability prediction models in the community setting, but we could not identify any references. However, evaluating if the introduction of a prediction model changes care, e.g. increases physical therapy interventions, and improves outcome, e.g. reduced incidence of disability, will be an important future investigation.

### Limitations

Some limitations should be noted. Although both authors screened the included studies independently, titles and abstracts were only screened by one author. However, we included a large number of studies and model which makes it likely that we have a sample that is representative from population of studies.. One author collected the data, and although this process was double-checked, some data abstraction mistakes may have been made. Further, we inferred the performance of the models based on the distribution (median, interquartile range and range), but these should not be considered pooled results. The models, and their evaluation, differed substantially from each other and we did not consider it relevant nor appropriate to perform a meta-analysis.

## Conclusion

Currently available models for the prediction of future functional status in older persons have a low to moderate performance on average. Though multivariable models perform slightly better than univariable models, high risks of bias in the evaluations of prediction models do not allow for any firm conclusions. There is currently no model that can be recommended for implementation in practice, but frailty models appear to be the most promising. Substantial improvements can be made in the methodology of developing and validating prediction models for disability in the community setting.

## Supplementary Information

**Additional File 1.** Prediction Models for Functional Status in Community Dwelling Older Adults: Review Protocol, Search Strategy and Prisma Checklist.

## Data Availability

The dataset supporting the conclusions of this article is available upon request. Please contact bastiaan.vangrootven@kuleuven.be.
