# Supplementary material for: Prediction models for functional status in community dwelling older adults: a systematic review
Source: BMC Geriatr. 2022 May 30;22:465. doi: 10.1186/s12877-022-03156-7 (PMC9150308; doi:10.1186/s12877-022-03156-7)
Supplement: Supplementary file 1 — Additional File 1. Prediction Models for Functional Status in Community Dwelling Older Adults: Review Protocol, Search Strategy and Prisma Checklist. [file 12877_2022_3156_MOESM1_ESM.docx]

**Appendix**

**Prediction models for functional status in community dwelling older adults: protocol for a scoping review**

Bastiaan Van Grootven, Theo van Achterberg

Appendix 1. Review protocol pg 2

Appendix 2. Search strategy pg 5

Appendix 3. Prisma checklist pg 11

**Appendix 1. Review protocol**

**Review team**

Bastiaan Van Grootven, Theo van Achterberg.

***Funding sources/sponsors***

None

***Conflict of interest***

None

***Collaborators***

n/a

***Review question***

Which models have been developed and validated for for the prediction of functional status in community dwelling older adults?

What predictors have been used in the models?

How well do the models predict functional status?

What methods have been used for the development, updating and validation of the models?

**Searches**

We will search the Medline and EMBASE databases from inception up to present for eligible studies. After selecting the full text manuscripts, we will screen references lists and prospective citations (using Google Scholar) for eligible studies. The ICTPR portal will be searched for protocols and Web of Science for conference proceedings.

**Search string**

See Appendix 2.

***Conditions or domain being studies***

Our primary outcome of interest is functional status. Functional status will be defined as the ability to perform (instrumental) activities of daily living (ADL) or ability to mobilise. The outcome can include decline, maintenance, recovery or improvement in functional status. There is no defined endpoint. Physical performance measures will be considered relevant if the reported data relates to daily activities, e.g. ability to mobilise. Physical performance related to strength or speed will not be considered for inclusion.

***Participants***

Older adults, indicated by a mean sample age of 65 years or older, living at home.

***Exposure***

Models designed to predict functional status. The model must measure a single characteristic or a set of characteristics to predict a person’s individual prognosis and can include patient, outcome and care process factors. The models can be presented in several formats, e.g. as a statistical model, regression formula with coefficients, spreadsheet, web or electronic application, nomogram, score chart, table with predictors, graphical score chart, regression tree, or probability/survival by risk group.

***Comparator***

Not applicable

***Types of studies to be included***

We will include nested case-control studies, prospective and retrospective cohort studies (including database and registry studies), and secondary analyses of trials.

***Main outcome***

Model discrimination measured using concordance (c) statistics for binary outcomes or R2 for continuous outcomes, and model calibration.

***Additional outcomes***

Description of predictors and methods used in the prediction models.

***Data extraction (selection and coding)***

Search results will be uploaded in an Endnote library. One author will select studies independently in a two stage process: screening titles and abstracts, and reading full text manuscripts. A second author will verify the included studies? The author will not be blinded to the manuscripts’ citation information. Corresponding authors will be contacted if additional information is needed for the screening procedure. Reasons for exclusion will be recorded.

A data extraction manual will be developed. One author will collect the data. Corresponding authors will be contacted to resolve uncertainties.

The following data will be collected in an Excell database, in accordance with the CHARMS checklist (Critical Appraisal and Data Extraction for Systematic Reviews): citation, source of data, country, study design, setting, participant description, sample characteristics, study dates, outcome definition, follow-up, number and type of predictors, definition and method for measurement of predictors, timing of predictor measurement, handling of predictors in the modelling, number of participants and number of outcomes/events, calibration, discrimination, classification, methods used for testing model performance, final multivariable model results (regression coefficients, intercept, baseline survival, model performance), and model presentation.

*Moons KGM et al. Critical Appraisal and Data Extraction for Systematic Reviews of Prediction Modelling Studies: The CHARMS Checklist. PLoS Med 2014. 11(10): e1001744.*

***Risk of bias***

The PROBAST tool (Prediction model Risk Of Bias ASsessment Tool) will be used to assess the risk of bias for the participants, predictors, outcome and analysis for each model. A standardised questionnaire will be used to rate the risk of bias as ‘yes’, ‘probably yes’, ‘no’, ‘probably no’, or ‘no information’. An overall judgement is made as either low risk of bias, high risk of bias or unclear risk of bias. One author will assess the risk of bias. Corresponding authors will be contacted to resolve uncertainties.

*Wolff RF, et al. PROBAST: A Tool to Assess the Risk of Bias and Applicability of Prediction Model Studies. Ann Intern Med 2019. 170(1): 51-58.*

***Strategy for data synthesis***

A narrative synthesis and summary tables will describe the study characteristics, risk of bias and findings of the studies. Discrimination will be reported by the concordance (c)-statistic. If not reported, we will try to derive the parameter from the available data. For the calibration, we will primarily use the total number of observed and expected events (across risk strata), but also consider the calibration slope, calibration in large or the Hosmer-Lemeshow test.

Funnel plots will be used to visualise the discrimination and calibration measures.

A meta-analysis will not be performed.

***Analysis of subgroups or subsets***

No subgroups will be specified in advance.

**Appendix 2. Search strategy**

**Search string for Medline (Ovid SP) Hits**

1. Functional-status.ab,ti. 24748

2. Functional-decline.ab,ti. 5087

3. Functioning.ab,ti. 176584

4. Self care.sh. 35112

5. Self-care.ab,ti. 17653

6. Activities of Daily Living.sh. 69651

7. Activities-of-daily-living.ab,ti. 25972

8. (Daily adj3 limitation*).ab,ti. 1212

9. (Limitation* adj3 activit*).ab,ti. 6002

10. ADL.ab,ti. 10075

11. Mobility limitation.sh. 5146

12. Mobility.ab,ti. 126653

13. Walking.ab,ti. 69620

14. Ambulation.ab,ti. 10728

15. Community participation.sh. 18093

16. (Participation adj3 community).ab,ti. 4699

17. (Participation adj3 public).ab,ti. 1198

18. (Community adj3 involvement*).ab,ti. 2439

19. Life-space.ab,ti. 356

20. Physical functional performance.sh. 2257

21. (Physical adj3 performance).ab,ti. 12063

22. (Physical adj3 function*).ab,ti. 36268

23. (Functional adj3 performance).ab,ti. 6377

24. (physical adj3 activity).ab,ti. 110514

25. Community-dwelling.ab,ti. 24405

26. (Living adj3 home).ab,ti. 4071

27. Primary health care.sh. 86923

28. Primary-health-care.ab,ti. 20973

29. (Primary adj3 healthcare).ab,ti. 7143

30. (Primary adj3 care).ab,ti. 137674

31. Home care services.sh. 35441

32. Home-care-service*.ab,ti. 1804

33. (Home adj3 care).ab,ti. 29847

34. (Home adj3 service*).ab,ti. 6359

35. (Home adj3 health).ab,ti. 8891

36. Community health services.sh. 32733

37. (Community adj3 care).ab,ti. 19729

38. (Community adj3 service*).ab,ti. 16294

39. (Community adj3 health*).ab,ti. 51040

40. Community health nursing.sh. 19738

41. Community-health-nursing.ab,ti. 813

42. (Community adj3 nursing).ab,ti. 4511

43. Independent Living.sh. 9313

44. Independent-living.ab,ti. 2621

45. Stratification.ab,ti. 64716

46. ROC Curve.sh. 67929

47. ROC-curve.ab,ti. 27726

48. Discrimination.ab,ti. 112021

49. Discriminate.ab,ti. 59534

50. c-statistic.ab,ti. 5215

51. c-index.ab,ti. 4259

52. Area-under-the-curve.ab,ti. 56673

53. AUC.ab,ti. 75606

54. Calibration.ab,ti. 61702

55. Indices.ab,ti. 149096

56. Algorithm.ab,ti. 159023

57. Multivariable.ab,ti. 112873

58. Decision support techniques.sh. 22114

59. Decision-support-technique*.ab,ti. 25

60. Predict*.ab,ti. 1515285

61. Prognos*.ab,ti. 598570

62. Nomograms.sh. 5799

63. Nomogram.ab,ti. 8422

64. Score-chart.ab,ti. 129

65. Regression-tree.ab,ti. 2471

66. Big data.sh. 2029

67. Big-data.ab,ti. 6090

68. Machine learning.sh. 22766

69. Machine-learning.ab,ti. 33409

70. 1 or 2 or 3 or 4 or 5 or 6 or 7 or 8 or 9 or 10 or 11 or 12 642484

or 13 or 14 or 15 or 16 or 17 or 18 or 19 or 20 or 21 or 22

or 23 or 24

71. 25 or 26 or 27 or 28 or 29 or 30 or 31 or 32 or 33 or 34 or 35 341167

or 36 or 37 or 38 or 39 or 40 or 41 or 42 or 43 or 44

72. 45 or 46 or 47 or 48 or 49 or 50 or 51 or 52 or 53 or 54 or 55 2580448

or 56 or 57 or 58 or 59 or 60 or 61 or 62 or 63 or 64 or 65 or

66 or 67 or 68 or 69

73. 70 and 71 and 72 6672

**Search string for Embase Hits**

1. Functional-status:ab,ti 42292
2. Functional-decline:ab,ti 9106
3. Functioning:ab,ti 272053
4. Self care/exp 93270
5. Self-care:ab,ti 29734
6. Daily life activity/exp 102690
7. ADL disability/exp 3879
8. Activities-of-daily-living:ab,ti 42001
9. Daily limitation*:ab,ti 33
10. Limitation* activit*:ab,ti 56
11. ADL:ab,ti 19159
12. Walking difficulty/exp 14312
13. Mobility:ab,ti. 186907
14. Walking:ab,ti 113100
15. Ambulation:ab,ti 18942
16. Community participation/exp 3769
17. Community participation:ab,ti 3889
18. Public participation:ab,ti 1034
19. Community involvement:ab,ti 1957
20. Life space:ab,ti 537
21. Physical performance/exp 110328
22. Physical performance:ab,ti 15331
23. Physical function*:ab,ti 44620
24. Functional performance:ab,ti 15331
25. Physical activity:ab,ti 17136
26. Community dwelling:ab,ti 35265
27. Living home:ab,ti 88
28. Primary health care/exp 189378
29. Primary health care:ab,ti 28037
30. Primary healthcare:ab,ti 9746
31. Primary care:ab,ti 174381
32. Home care:ab,ti 23792
33. Home care service*:ab,ti 592
34. Home care/exp 83507
35. Home service*:ab,ti 1007
36. Home health:ab,ti 7849
37. Community care/exp 133706
38. Community care:ab,ti 6133
39. Community service*:ab,ti 6133
40. Community health*:ab,ti 33965
41. Community health nursing/exp 27776
42. Community health nursing:ab,ti 880
43. Community nursing:ab,ti 2022
44. Independent Living/exp 6206
45. Independent living:ab,ti 4203
46. Stratification:ab,ti 126158
47. receiver operating characteristic/exp 168256
48. ROC curve:ab,ti 59354
49. Discrimination:ab,ti 157635
50. Discriminate:ab,ti 87718
51. C statistic:ab,ti 10523
52. C index:ab,ti 9193
53. Area under the curve:ab,ti 82291
54. AUC:ab,ti 166598
55. Calibration:ab,ti 102531
56. Indices:ab,ti 222152
57. Algorithm:ab,ti 283737
58. Multivariable:ab,ti 207584
59. Decision support system/exp 30008
60. Decision support technique*:ab,ti 32
61. Predict*:ab,ti 2454622
62. Prognos*:ab,ti 1066080
63. Nomogram/exp 16227
64. Nomogram:ab,ti 16194
65. Score chart:ab,ti 296
66. Regression tree:ab,ti 3856
67. Big data/exp 4145
68. Big data:ab,ti 11023
69. Machine learning/exp 298236
70. Machine learning:ab,ti 61961
71. 1 or 2 or 3 or 4 or 5 or 6 or 7 or 8 or 9 or 10 or 11 1062985

or 12 or 13 or 14 or 15 or 16 or 17 or 18 or 19 or 20

or 21 or 22 or 23 or 24 or 25

1. 26 or 27 or 28 or 29 or 30 or 31 or 32 or 33 or 34 525970

or 35 or 36 or 37 or 38 or 39 or 40 or 41 or 42 or 43

or 44 or 45

1. 46 or 47 or 48 or 49 or 50 or 51 or 52 or 53 or 54 4348195

or 55 or 56 or 57 or 58 or 59 or 60 or 61 or 62 or 63

or 64 or 65 or 66 or 67 or 68 or 69 or 70

1. 71 and 72 and 73 9535

**Appendix 3. Prisma checklist**

| **Section and Topic** | **Item #** | **Checklist item** | **Location where item is reported** |
| --- | --- | --- | --- |
| **TITLE** | | |  |
| Title | 1 | Identify the report as a systematic review. | 1 |
| **ABSTRACT** | | |  |
| Abstract | 2 | See the PRISMA 2020 for Abstracts checklist. | 3 |
| **INTRODUCTION** | | |  |
| Rationale | 3 | Describe the rationale for the review in the context of existing knowledge. | 4 |
| Objectives | 4 | Provide an explicit statement of the objective(s) or question(s) the review addresses. | 4 |
| **METHODS** | | |  |
| Eligibility criteria | 5 | Specify the inclusion and exclusion criteria for the review and how studies were grouped for the syntheses. | 5 |
| Information sources | 6 | Specify all databases, registers, websites, organisations, reference lists and other sources searched or consulted to identify studies. Specify the date when each source was last searched or consulted. | 6 |
| Search strategy | 7 | Present the full search strategies for all databases, registers and websites, including any filters and limits used. | Appendix 2 |
| Selection process | 8 | Specify the methods used to decide whether a study met the inclusion criteria of the review, including how many reviewers screened each record and each report retrieved, whether they worked independently, and if applicable, details of automation tools used in the process. | 6 |
| Data collection process | 9 | Specify the methods used to collect data from reports, including how many reviewers collected data from each report, whether they worked independently, any processes for obtaining or confirming data from study investigators, and if applicable, details of automation tools used in the process. | 6 |
| Data items | 10a | List and define all outcomes for which data were sought. Specify whether all results that were compatible with each outcome domain in each study were sought (e.g. for all measures, time points, analyses), and if not, the methods used to decide which results to collect. | 7 |
|  | 10b | List and define all other variables for which data were sought (e.g. participant and intervention characteristics, funding sources). Describe any assumptions made about any missing or unclear information. | 7 |
| Study risk of bias assessment | 11 | Specify the methods used to assess risk of bias in the included studies, including details of the tool(s) used, how many reviewers assessed each study and whether they worked independently, and if applicable, details of automation tools used in the process. | 7 |
| Effect measures | 12 | Specify for each outcome the effect measure(s) (e.g. risk ratio, mean difference) used in the synthesis or presentation of results. | 7 |
| Synthesis methods | 13a | Describe the processes used to decide which studies were eligible for each synthesis (e.g. tabulating the study intervention characteristics and comparing against the planned groups for each synthesis (item #5)). | 7,8 |
|  | 13b | Describe any methods required to prepare the data for presentation or synthesis, such as handling of missing summary statistics, or data conversions. | 7,8 |
|  | 13c | Describe any methods used to tabulate or visually display results of individual studies and syntheses. | 8 |
|  | 13d | Describe any methods used to synthesize results and provide a rationale for the choice(s). If meta-analysis was performed, describe the model(s), method(s) to identify the presence and extent of statistical heterogeneity, and software package(s) used. | 7,8 |
|  | 13e | Describe any methods used to explore possible causes of heterogeneity among study results (e.g. subgroup analysis, meta-regression). | 8 |
|  | 13f | Describe any sensitivity analyses conducted to assess robustness of the synthesized results. | 8 |
| Reporting bias assessment | 14 | Describe any methods used to assess risk of bias due to missing results in a synthesis (arising from reporting biases). | n/a |
| Certainty assessment | 15 | Describe any methods used to assess certainty (or confidence) in the body of evidence for an outcome. | n/a |
| **RESULTS** | | |  |
| Study selection | 16a | Describe the results of the search and selection process, from the number of records identified in the search to the number of studies included in the review, ideally using a flow diagram. | 8 |
|  | 16b | Cite studies that might appear to meet the inclusion criteria, but which were excluded, and explain why they were excluded. | 8 |
| Study characteristics | 17 | Cite each included study and present its characteristics. | 10 – 13 |
| Risk of bias in studies | 18 | Present assessments of risk of bias for each included study. | 14 |
| Results of individual studies | 19 | For all outcomes, present, for each study: (a) summary statistics for each group (where appropriate) and (b) an effect estimate and its precision (e.g. confidence/credible interval), ideally using structured tables or plots. | 15 -17 |
| Results of syntheses | 20a | For each synthesis, briefly summarise the characteristics and risk of bias among contributing studies. | 15 -17 |
|  | 20b | Present results of all statistical syntheses conducted. If meta-analysis was done, present for each the summary estimate and its precision (e.g. confidence/credible interval) and measures of statistical heterogeneity. If comparing groups, describe the direction of the effect. | 15 -17 |
|  | 20c | Present results of all investigations of possible causes of heterogeneity among study results. | 15 -17 |
|  | 20d | Present results of all sensitivity analyses conducted to assess the robustness of the synthesized results. | 15 -17 |
| Reporting biases | 21 | Present assessments of risk of bias due to missing results (arising from reporting biases) for each synthesis assessed. | n/a |
| Certainty of evidence | 22 | Present assessments of certainty (or confidence) in the body of evidence for each outcome assessed. | n/a |
| **DISCUSSION** | | |  |
| Discussion | 23a | Provide a general interpretation of the results in the context of other evidence. | 18 – 20 |
|  | 23b | Discuss any limitations of the evidence included in the review. | 20 |
|  | 23c | Discuss any limitations of the review processes used. | 20 |
|  | 23d | Discuss implications of the results for practice, policy, and future research. | 19 |
| **OTHER INFORMATION** | | |  |
| Registration and protocol | 24a | Provide registration information for the review, including register name and registration number, or state that the review was not registered. | Appendix 1 |
|  | 24b | Indicate where the review protocol can be accessed, or state that a protocol was not prepared. | Appendix 1 |
|  | 24c | Describe and explain any amendments to information provided at registration or in the protocol. | Appendix 1 |
| Support | 25 | Describe sources of financial or non-financial support for the review, and the role of the funders or sponsors in the review. | n/a |
| Competing interests | 26 | Declare any competing interests of review authors. | 1 |
| Availability of data, code and other materials | 27 | Report which of the following are publicly available and where they can be found: template data collection forms; data extracted from included studies; data used for all analyses; analytic code; any other materials used in the review. | n/a |
